# Supplementary material for: Nicotine Induces Polyspermy in Sea Urchin Eggs through a Non-Cholinergic Pathway Modulating Actin Dynamics
Source: Cells. 2019 Dec 25;9(1):63. doi: 10.3390/cells9010063 (PMC7016604; doi:10.3390/cells9010063)
Supplement: Supplementary file 1 [file cells-09-00063-s001.zip › supple.pdf]

Supplementary Data.

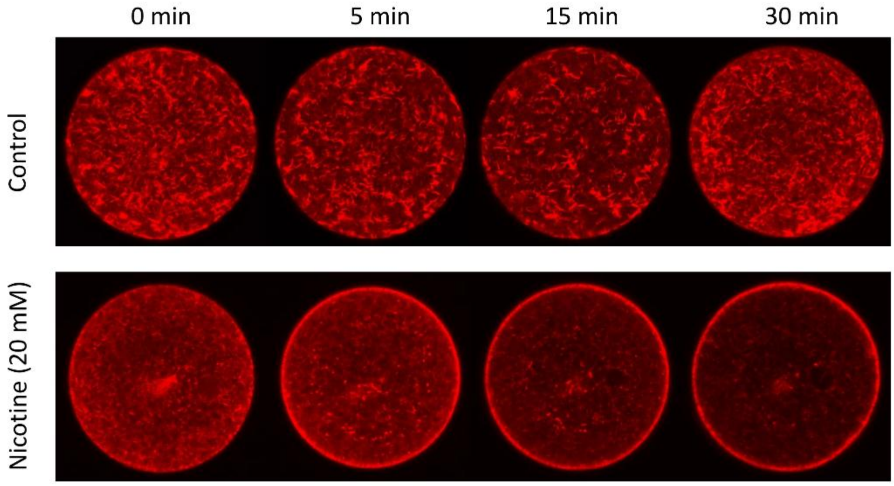

Figure S1. A high dose of nicotine induces massive polymerization of cortical actin cytoskeleton in sea urchin eggs within a few minutes of incubation. *P. lividus eggs*, microinjected with AlexaFluor 568–phalloidin (10 μM, pipette concentration), were incubated in the presence or absence (control) of 20 mM nicotine, and the changes of F-actin were monitored on the equatorial plane of the same individual eggs with a Zeiss LSM 510 META laser scanning confocal microscope (Jena, Germany).

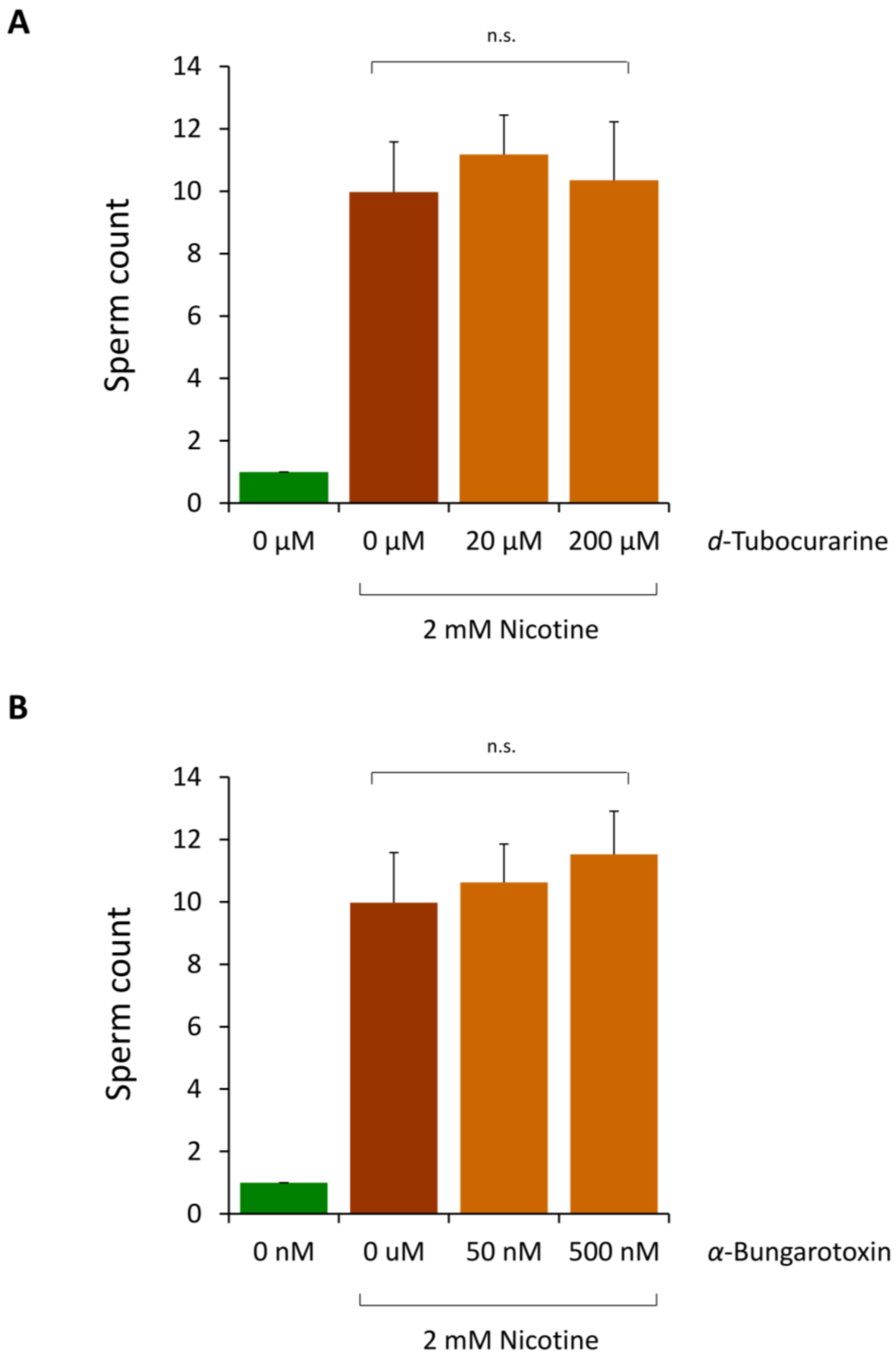

Figure S2. Competitive antagonists of nAChR do not inhibit nicotine-induced polyspermic fertilization. *P. lividus* eggs were pretreated with d-tubocurarine (A) or α-bungarotoxin (B) for 5 min prior to the exposure to (-)nicotine (5 min). The eggs (n=40 for each condition) were fertilized with Hoechst 33342-prestained sperm and the numbers of egg-incorporated sperm were counted 10 min after insemination. Differences statistically not significant (one-way ANOVA) were marked with "n.s."

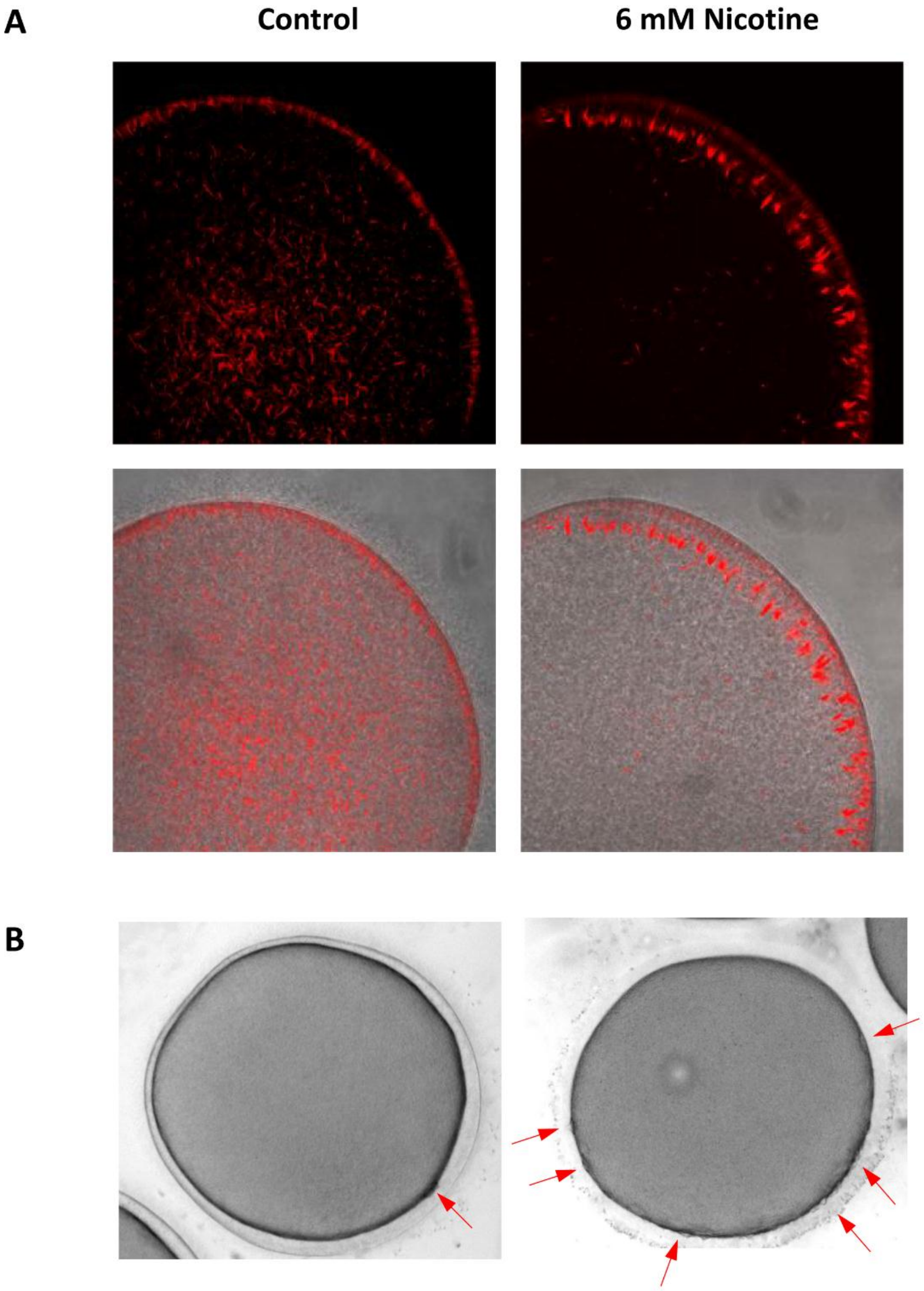

Figure S3. Nicotine induces both cortical F-actin changes and polyspermy in starfish eggs. Mature eggs of starfish (*Astropecten aranciacus*) were incubated for 40 min in the presence or absence (control) of 6 mM nicotine. (A) F-actin stained by microinjected AlexaPhalloidin (top panel). Bottom panel: merged view with the transmission image. (B) About 5 min after fertilization, multiple fertilization cones were formed in the eggs pretreated with nicotine (red arrows), whereas the control eggs show only one.   .

Video S1. Movements of F-actin in the control eggs at fertilization. *P. lividus* eggs microinjected with Alexa-Phalloidin were fertilized to monitor movement of the cortical actin filaments by confocal microscopy.

Video S2. **Movements of F-actin in the nicotine-pretreated (20 mM, 5 min) eggs at fertilization**.
